# Analyzing COVID-19 progression with Markov multistage models: insights from a Korean cohort

**DOI:** 10.1186/s44342-024-00035-y

**Published:** 2025-01-27

**Authors:** Frank Aimee Rodrigue Ndagijimana, Taesung Park

**Affiliations:** 1 Interdisciplinary Program in Bioinformatics, Seoul National University, Seoul, Republic of Korea; 2 Department of Statistics, Seoul National University, Seoul, Republic of Korea

**Keywords:** COVID-19 progression, Markov multistate model, Transition intensity, Comorbidity, Severity state

## Abstract

**Background:**

Understanding the progression and recovery process of COVID-19 is crucial for guiding public health strategies and developing targeted interventions. This longitudinal cohort study aims to elucidate the dynamics of COVID-19 severity progression and evaluate the impact of underlying health conditions on these transitions, providing critical insights for more effective disease management.

**Methods:**

Data from 4549 COVID-19 patients admitted to Seoul National University Boramae Medical Center between February 5th, 2020, and October 30th, 2021, were analyzed using a 5-state continuous-time Markov multistate model. The model estimated instantaneous transition rates between different levels of COVID-19 severity, predicted probabilities of state transitions, and determined hazard ratios associated with underlying comorbidities.

**Results:**

The analysis revealed that most patients stabilized in their initial state, with 72.2% of patients with moderate symptoms remaining moderate. Patients with hypertension had a 67.6% higher risk of progressing from moderate to severe, while those with diabetes had an 89.9% higher risk of deteriorating from severe to critical. Although transition rates to death were low early in hospitalization, these comorbidities significantly increased the likelihood of worsening conditions.

**Conclusion:**

This study highlights the utility of continuous-time Markov multistate models in assessing COVID-19 severity progression among hospitalized patients. The findings indicate that patients are more likely to recover than to experience worsening conditions. However, hypertension and diabetes significantly increase the risk of severe outcomes, underscoring the importance of managing these conditions in COVID-19 patients.

**Supplementary Information:**

The online version contains supplementary material available at 10.1186/s44342-024-00035-y.

## Introduction

COVID-19, or coronavirus disease 2019, is a highly contagious illness primarily impacting the respiratory system. It is caused by the novel severe acute respiratory syndrome coronavirus 2 (SARS-CoV-2), an enveloped, single-stranded positive-sense RNA virus classified within the *Betacoronavirus* genus of the *Coronaviridae* family [1, 2]. This virus first emerged in December 2019 in Wuhan, the capital of Hubei province in China [3]. The spread of this virus worldwide led to unprecedented changes in daily lifestyle that significantly influenced the global economy and led to profound changes in social life and public health [4–7]. SARS-CoV-2 is predominantly transmitted through respiratory secretions, particularly droplets expelled during coughing, sneezing, and talking. Moreover, transmission can occur through personal contact and contaminated surfaces or fomites, especially in settings where non-pharmaceutical interventions (NPIs), including hand hygiene, the proper use of masks, and appropriate social distancing, are not consistently implemented [8]. Furthermore, airborne transmission of smaller aerosolized droplets is possible, particularly in enclosed or poorly ventilated environments [9, 10]. International air travel has significantly accelerated the spread of the virus, exacerbating its cross-border transmission [11]. Consequently, the World Health Organization (WHO) declared COVID-19 a public health emergency of international concern on January 30, 2020 [12], and elevated it to a global pandemic on March 11, 2020 [13]. As of September 2024, the World Health Organization (WHO) reports more than 776 million confirmed cases and over 7 million deaths globally [14]. COVID-19 presents a wide range of clinical manifestations, with symptoms that can vary between patients and fluctuate over time, ranging from mild to severe, while some individuals may remain asymptomatic [15].

Reflecting the varying clinical manifestations of COVID-19, the World Health Organization (WHO) has established comprehensive guidelines based on clinical symptoms to categorize the severity of COVID-19 infections, facilitating optimal patient management and the efficient allocation of medical resources [16]. This classification system categorizes cases of infection into five distinct states: asymptomatic (presymptomatic), mild, moderate, severe, and critical. Asymptomatic individuals test positive for SARS-CoV-2 virus but do not show any symptoms of the infection. Mild cases typically present with symptoms like fever, cough, and loss of taste or smell without respiratory distress. Moderate cases show lower respiratory issues with oxygen saturation (SpO2) at 94% or higher on room air. Severe cases include more serious respiratory problems, such as SpO2 below 94% or a respiratory rate over 30 breaths per minute. Critical cases involve life-threatening conditions like respiratory failure, septic shock, or multiple organ dysfunction. The diverse clinical manifestations of COVID-19 are influenced by demographic factors such as age and sex, along with pre-existing chronic health conditions. Research consistently highlights that older adults and those with chronic illnesses such as hypertension and diabetes are at notably higher risk for adverse disease outcomes including symptom aggravation, admission to the intensive care unit (ICU), and death [17–19].

The COVID-19 pandemic has placed an unprecedented burden on global public health, overwhelming healthcare systems, depleting critical medical resources, and leading to substantial morbidity and mortality. In response to these challenges, predictive models, including machine learning algorithms and mathematical simulations, have become crucial tools for guiding clinical decision-making, forecasting disease progression and evaluating the effectiveness of interventions to mitigate the pandemic’s impact. Among these tools, the open-source online COVID-19 Community Mortality Risk Prediction (CoCoMoRP) model leverages logistic regression to predict mortality risk in South Korea, providing support to healthcare providers and policymakers [20]. Machine learning algorithms, including eXtreme Gradient Boosting, K-Nearest Neighbor, Random Forest, bagged-CART, and LogitBoost algorithms were also employed to predict ICU admission, mortality, and length of stay in hospitalized COVID-19 patients [21]. Moreover, deep learning approaches integrating X-ray information and clinical data have been proposed to enhance COVID-19 detection, severity classification, and outcome prediction [22–24]. Other studies have aimed to identify risk factors for COVID-19 and examine their associations with disease outcomes. For example, numerous studies have investigated the association between pre-existing comorbidities and mortality, disease severity in patients diagnosed with COVID-19 [25, 26].

Although existing studies have significantly enhanced our understanding of COVID-19, the temporal progression of COVID-19 severity remains unclear. Much of the current research focuses on predicting endpoints such as recovery or mortality, with limited attention to dynamic transitions between the severity states. The mechanisms driving these transitions and the role of comorbidities in shaping patients’ trajectories, remain poorly understood. Therefore, a nuanced understanding of how and when individuals progress from one state of severity to another is crucial for shaping proactive, real-time interventions that address disease progression in its intermediate stages rather than solely forecasting terminal endpoints. To study these transitions comprehensively, several studies have been proposed based on Markov models and multistate models which are essential tools for analyzing stochastic processes involving state transitions. These models are governed by the Markov property, which states that the future state solely depends on the current state of the process. Markov models are simpler and ideal for studying population-level dynamics, such as disease transmission and intervention outcomes. On the other hand, multistate models extend this framework by incorporating multiple interconnected states, enabling detailed analyses of complex systems while still adhering to the Markov property. In the context of COVID-19, these models have been applied to study disease transmission, patient outcomes, and intervention strategies. For example, a study by Noh et al. investigated the phase shifts in SARS-Cov-2 sub-lineages by employing a Markov switching model [27]. Another study by Wang and Mustafa applied a data-driven Markov model to evaluate COVID-19 transmission dynamics and interventions [28]. Hazard et al. employed multistate models with four states to study the progress of patients admitted to the ICU [29]. Al-Zoughool et al. employed a stochastic Continuous-Time Markov Chain (CTMC) model with eight states to simulate the transmission dynamics of SARS-CoV-2 [30]. The analysis focused on evaluating various hypothetical lockdown scenarios to determine the optimal timing and duration of lockdown measures that could effectively control new infections and reduce hospitalizations.

While these Markov multistate models have been applied to several COVID-19 studies, none have been applied to the Korean population. Since it is well known that the progress of COVID-19 would differ depending on race and national pandemic prevention policies [31, 32], it would be important to study the progression of COVID-19 in the South Korean population. Thus, the present study employs Markov multistate models to analyze the progression of disease severity within a longitudinal cohort of hospitalized COVID-19 patients from a Korean hospital. In particular, we employed a continuous-time Markov multistate model to analyze a longitudinal dataset of COVID-19 patients hospitalized at Seoul National University (SNU) Boramae Medical Center in Seoul, South Korea. The primary aim was to investigate the patterns of disease progression and recovery over time. The model facilitated the estimation of transition intensities, computation of transition probabilities, and determination of mean sojourn times. Furthermore, given that diabetes and hypertension are widely recognized as key contributors to adverse COVID-19 outcomes [33–35], this study also aimed to examine how these conditions, as potential covariates, influence the transitions of disease severity. The findings of this study are expected to inform targeted interventions, optimize resource allocation, and facilitate timely responses to COVID-19, thereby contributing to improved patient outcomes. To the best of our knowledge, this is the first study to employ continuous-time Markov multistate models to investigate the progression of COVID-19 severity in South Korea.

## Materials and methods

### Data acquisition and preprocessing

This study collected data from 4587 COVID-19 patients admitted at Seoul National University (SNU) Boramae Medical Center from February 5, 2020, to October 30, 2021. The demographic and clinical information of the patients were recorded upon admission, and patients were subjected to daily monitoring of disease severity in accordance with the guidelines established by the World Health Organization (WHO) [16]. The severity of illness was classified into five states based on clinical manifestations: mild (State 1), moderate (State 2), severe (State 3), critical (State 4), or death (State 5), as outlined in Supplementary Table S1. The current study defined a shift in severity state as a state transition. The preprocessing step involved the exclusion of 38 patients with incomplete clinical records, resulting in a final dataset of 4549 patients.

## Markov multistate analysis

### Multistate model framework

**Fig. 1:**
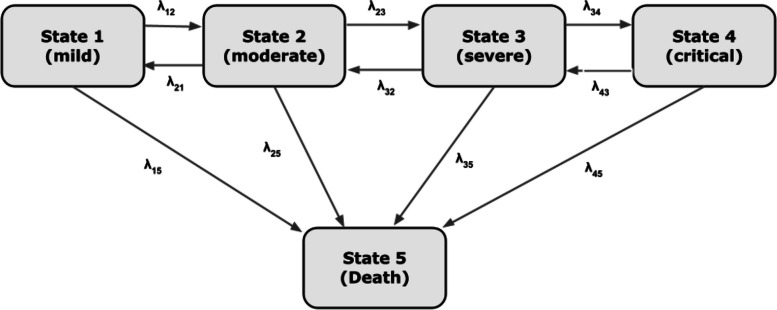
Proposed 5-state model for examining disease progression among COVID-19 patients, SNU Boramae Medical Center

In this study, a continuous-time first-order Markov multistate model was employed to study the transitions between different states of severity of COVID-19, as illustrated in Fig. 1. In the proposed model, a patient in State 1 may either remain in this state, progress to State 2 or die. An individual in State 2 can either remain in this state, recover to State 1, or advance to either State 3 or State 5. A patient in State 3 may recover to State 2, deteriorate to State 4, or die. Additionally, patients in State 4 may either recover to State 3 or experience death. Once a patient transitions into State 5, no further transitions are permissible, thereby designating this state as the absorbing state within the model.

### Estimation of transition intensities

Multistate models are governed by transition intensity functions that characterize the instantaneous risk of moving from one state to another. In the context of a continuous-time Markov model, the transition intensity from state *i to* state *j*, denoted by ${\uplambda }_{\mathit{ij}}(t)$, represents the instantaneous rate at which patients in state *i* transition to state *j* within a time interval $\Delta t$ given that a patient is in state *i* at time $t$. This is mathematically expressed as:

1 $${\uplambda }_{\mathit{ij}}(t)=\underset{\Delta t\to 0}{\text{lim}}\frac{P\left(X\left(t+\Delta t\right)=j \right| X\left(t\right)=i)}{\Delta t}$$

where ${\uplambda }_{\mathit{ij}}(t)$ is the transition intensity from state *i* to state *j*, and $P\left(X\left(t+\Delta t\right)=j \right| X\left(t\right)=i)$ denotes the probability of transition from state $i$ to state $j$ over the time interval $\Delta t$. The collection of transition intensities constitutes the transition intensity matrix $Q$, which encompasses all possible transition rates among the various states.

Transition intensities between states were estimated by maximizing the likelihood function based on each patient’s observed transitions over time. For a patient in state *i*, the likelihood of transitioning to state *j* during the time interval $\Delta t={[\text{t}}_{i-1,}{\text{t}}_{i}]$ is represented by the transition probability $P(X({t}_{i})=j\mid X({t}_{i-1})=i,Q)$ where $Q$ denotes the matrix of transition intensities. The overall likelihood for a dataset with N patients is expressed as:

2 $$\prod_{i=1}^{N}\prod_{{[\text{t}}_{i-1,}{\text{t}}_{i}]}P(X({t}_{i})=j\mid X({t}_{i-1})=i,Q)$$

### Transition probability matrix and mean sojourn times

The transition probability matrix, $P\left(t\right)$ is a matrix that provides the probabilities of being in a state $j$ at time $t$ after being in state $i$ at time $t-1$. Each element ${P}_{ij}(t)$ of the matrix represents the probability that a patient in state $i$ at time 0 will be in state $j$ after time $t$. The transition probability matrix is obtained by taking the matrix exponential of the transition intensity matrix, expressed as the following:

3 $$P\left(t\right)=\text{Exp}\left(tQ\right)$$

The mean sojourn times indicate the average time a patient is expected to remain in each state before transitioning to another. For any state $i$, the mean sojourn time, denoted as${\tau }_{i}$, is calculated as the inverse of the total transition rate out of that state. This is expressed mathematically as the following:

4 $${\tau }_{i}=\frac{1}{\sum_{j\ne i}{\lambda }_{ij}}$$

### Incorporating comorbidities in the model

Patient-specific covariates, diabetes, and hypertension were incorporated into the model to examine the impact of these comorbidities on state transitions. These covariates were chosen based on literature-based evidence of their association with adverse outcomes of COVID-19, including high severity and mortality. Numerous studies have consistently identified diabetes and hypertension as critical risk factors for adverse outcomes of the infection. For example, a study examining chronic diseases in Mexican COVID-19 patients demonstrated that both diabetes and hypertension significantly increased case-fatality rates and contributed to higher disease severity levels among hospitalized individuals [33]. Another study assessing risk factors for COVID-19 mortality identified hypertension and diabetes, alongside age and geographic region, as primary determinants of severe outcomes in COVID-19 [34]. Furthermore, a systematic review and meta-analysis of risk factors for COVID-19 severity and mortality confirmed that chronic conditions, including diabetes and hypertension, significantly increased the risk of severe outcomes, alongside advanced age and cardiovascular diseases [35]. Grounded in well-documented evidence, we incorporated diabetes and hypertension as covariates to critically evaluate their role in driving state transitions and determining the progression of COVID-19 disease.

The transition intensities ${\lambda }_{ij}$ between states are modeled as a function of these covariates, using a proportional hazards framework. The adjusted transition intensity is expressed as the following:

$${\lambda }_{ij}\left(t|Z\right)= {\lambda }_{ij}^{0}\left(\text{t}\right)\text{ exp}({\beta }_{ij}^{T}Z)$$

where ${\lambda }_{ij}^{0}\left(\text{t}\right)$ indicates the baseline transition intensity from state $i$ to state $j$, ${\beta }_{ij}$ is a vector of coefficients representing the effects of the covariates and $Z$ is a vector of covariates.

All analyses were conducted utilizing the msm package [36], which is available in the free software R (version 4.3.3; https://www.r-project.org).

## Results

### Data characteristics

**Fig. 2:**
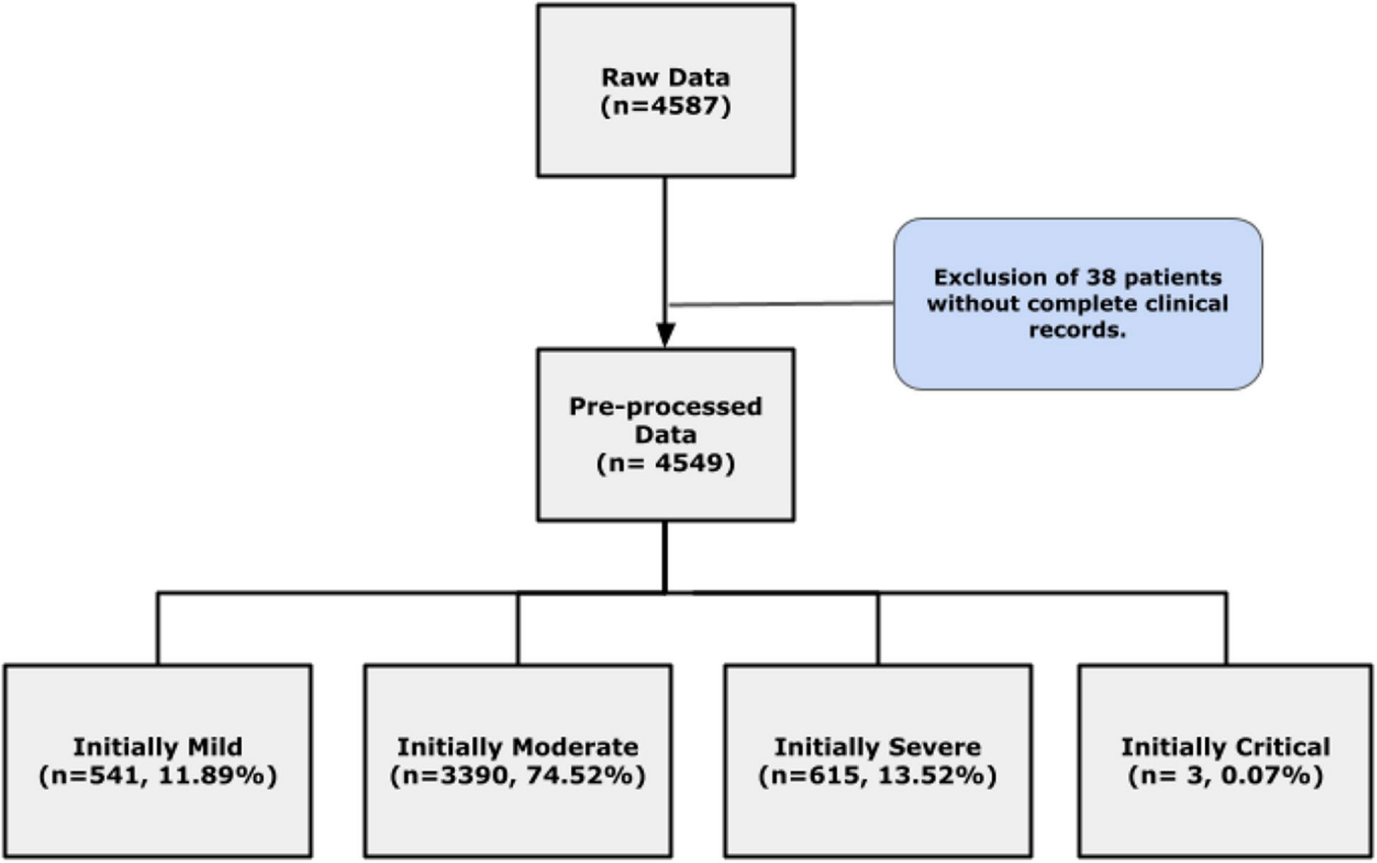
Pre-processing and composition of SNU Borame Hospital COVID-19 cohort

The data preprocessing yielded a dataset of 4549 patients prepared for analysis as illustrated in Fig. 2. At the time of admission, 541 patients (11.89%) presented with mild symptoms, while 3390 patients (74.52%) exhibited moderate symptoms, and 615 patients (13.52%) were classified as having severe symptoms. Additionally, 3 patients (0.07%) were categorized as having critical symptoms of COVID-19. The ages of the participants ranged from 18 to 101 years, with a median age of 53 (Table 1). Regarding gender distribution, 2,448 patients (53.81%) were female, compared to 2,105 patients (46.19%) who were male, indicating a marginally higher representation of females. Among the recorded comorbidities, hypertension was the most prevalent, affecting 27.59% of patients, followed by diabetes at 14.9% (Table 1). Other concurrent diseases included dementia (5.34%), malignant neoplasms (4.37%), heart failure (3.72%), asthma (2.02%), chronic liver disease (1.38%), chronic cardiac disease (1.32%), chronic neurological disorders (1.06%), chronic kidney disease (0.97%), chronic obstructive pulmonary disease (0.37%), and chronic hematologic disease (0.35%).

**Table 1:** Demographic and clinical characteristics of Borame Hospital COVID-19 cohort

Characteristics	Values				
Demographics					
	Min	Mean (SD)	Median		Max
Age (years)	18.00	52.97 (17.57)	53.00		101
Body mass index (Kg/m^2^)	10.72	24.30 (4.15)	24.00		59.52
Gender	Male, *n* (%)		2101 (46.19)		
	Female, *n* (%)		2448 (53.81)		
Current smoker	Yes, *n* (%)		510 (11.21)		
	No, *n* (%)		4039 (88.79)		
Vital signs on admission					
	Min	Mean (SD)	Median		Max
Heart rate (beats/min)	42.00	89.68 (14.85)	89.00		143.00
Systolic blood pressure (mmHg)	66.00	131.18 (18.96)	130.00		222.00
Body temperature (^o^C)	33.80	37.04 (0.80)	36.9		40.40
Respiration rate (breaths/min)	14.00	19.66 (3.27)	20.00		98.00
Comorbidities					
	Yes, *n* (%)			No, *n* (%)	
Hypertension	1255 (27.59)			3294 (72.41)	
Diabetes mellitus	678 (14.90)			3871 (85.10)	
Dementia	243 (5.34)			4306 (94.66)	
Malignancy	199 (4.37)			4350 (95.63)	
Heart failure	169 (3.72)			4380 (96.28)	
Asthma	92 (2.02)			4457 (97.98)	
Chronic liver disease	63 (1.38)			4486 (98.62)	
Chronic cardiac disease	60 (1.32)			4489 (98.68)	
Chronic neurological disorder	48 (1.06)			4501 (98.94)	
Chronic kidney disease	44 (0.97)			4505 (99.03)	
Chronic obstructive pulmonary disease	17 (0.37)			4532 (99.63)	
Chronic hematological disease	16 (0.35)			4533 (99.65)	
Symptoms on admission					
	Yes *n* (%)			No *n* (%)	
Cough	2330 (51.22)			2219 (48.78)	
Sputum	1471 (32.34)			3078 (67.66)	
Headache	993 (21.83)			3556 (78.17)	
Sore throat	914 (20.09)			3635 (79.91)	
Myalgia	872 (19.17)			3677 (80.83)	
Vomiting/nausea	398 (8.75)			4151 (91.25)	
Diarrhea	305 (6.70)			4244 (93.30)	
Fatigue/malaise	292 (6.42)			4257 (93.58)	
Rhinorrhea	241 (5.30)			4308 (94.70)	

## Markov multistate analysis

### Frequencies of state transitions

The frequencies of transitions between consecutive observed states during the first 2 weeks of hospitalization are presented in Table 2. Most transitions involve patients remaining in the same state. The most frequent transitions were patients remaining in State 2 (21,899 cases), followed by those remaining in State 1 (3506 cases) and State 3 (2901 cases). Transitions to lower states of severity outnumbered transitions to relatively more severe states. Specifically, 837 patients improved from State 2 to State 1, compared to 717 who worsened to State 3. Transitions directly to State 5 were rare. Notably, only four deaths were recorded from State 3, and one death was recorded from State 4.

**Table 2:** Distribution of consecutive observed states among COVID-19 patients

Prior state	Next state				
	State 1	State 2	State 3	State 4	State 5
State 1	3506	491	127	0	0
State 2	837	21,899	717	8	1
State 3	139	460	2901	40	4
State 4	0	8	17	100	1
State 5	0	0	0	0	0

### Estimated transition intensities

Table 3 presents the primary results from our multistate analyses. For patients in State 1, the transition rate to State 2, *λ*_*12*_ is 0.168, identical to the rate of remaining in the same state (λ_*11*_ = 0.168). Individuals in State 2 are 1.13 times more likely to improve to a mild state (λ_*21*_ = 0.046) than to aggravate to a severe state (λ_*23*_ = 0.041), indicating a slight tendency toward improvement. Once in State 3, patients have a 13 times higher recovery rate to State 2 (λ_*32*_ = 0.195) compared to progression to State 4 (λ_*34*_ = 0.015), suggesting a greater likelihood of recovery even in severe conditions. Transition rates to State 5 were very low. For instance, the estimated transition rate from State 4 to 5 (*λ*_*45*_) was 0.011, while that from State 3 to 5 (λ_*35*_) was 0.001.

**Table 3:** Estimated transition intensity matrix of the multistate model among COVID-19 patients in the Boramae Hospital cohort study

Prior state	Next state				
	State 1	State 2	State 3	State 4	State 5
State 1	− 0.1681 (− 0.182, − 0.155)	0.1681 (0.155, 0.182)	0	0	2.649e^−09^ (2.343e^−293^, 2.993e^+275^)
State 2	0.046 (0.044, 0.0494)	− 0.087 (− 0.091, − 0.083)	0.041 (0.038, 0.044)	0	2.194e-07 (5.359e^−40^, 8.986e^25^)
State 3	0	0.195 (0.179, 0.211)	− 0.211 (− 0.228, − 0.196)	0.015 (0.011, 0.019)	0.001 (4.514e^−04^, 3.423e-^03^)
State 4	0	0	0.226 (0.015, 0.334)	− 0.237 (− 0.348, − 0.161)	0.011 (0.002, 0.082)
State 5	0	0	0	0	0

### Estimated transition probabilities and mean sojourn times

Using the estimated transition intensities, we calculated the probabilities of moving to different states after 7, 14, and 30 days following hospital admission as illustrated in Table 4. After 1 week, patients in State 1 were most likely to transition to State 2, with a probability of 0.551 (55.1%). Those in State 2 had a higher likelihood (72.2%) of remaining in the same state, with chances of improving to State 1 (15.2%) or worsening to State 3 at 12.1%. Patients in State 3 tended to stay in that state (31.3%) or improve to State 2 (57.4%).

**Table 4:** Estimated transition probabilities within 7, 14 and 30 days after hospitalization

Time and state	Estimated transition probability				
	State 1	State 2	State 3	State 4	State 5
7 days					
State 1	0.385	0.551	0.062	0.002	3e^−04^
State 2	0.152	0.722	0.121	0.005	0.001
State 3	0.082	0.574	0.313	0.025	0.006
State 4	0.036	0.345	0.369	0.208	0.041
State 5	0.000	0.000	0.000	0.000	1.000
14 days					
State 1	0.237	0.646	0.111	0.005	0.001
State 2	0.178	0.675	0.136	0.008	0.003
State 3	0.145	0.648	0.182	0.016	0.010
State 4	0.104	0.553	0.236	0.054	0.053
State 5	0.000	0.000	0.000	0.000	1.000
30 days					
State 1	0.186	0.664	0.136	0.008	0.005
State 2	0.183	0.663	0.138	0.009	0.007
State 3	0.179	0.657	0.140	0.009	0.015
State 4	0.166	0.625	0.139	0.011	0.060
State 5	0.000	0.000	0.000	0.000	1.000

By extending the observation period to 2 weeks, the probabilities indicated a strong tendency of patients initially in State 1 to transition to State 2 (64.6%). Those in State 2 mostly stabilized (67.5%), with slightly increased chances of transitioning to State 3 (13.6%) or back to State 1 (17.8%) compared to the first week. Patients admitted in State 3 tended to recover to State 2 (64.8%), while those in State 4 (critical state) showed equal probabilities of either transitioning to State 3 (23.6%) or remaining in State 4 (23.6%).

After about a month, the data demonstrated a consolidation trend for patients initially in State 1 and State 2, with a notable shift towards State 2. The probability of patients initially in State 1 transitioning to State 2 was 66.4%, while for those initially in State 2, it was 66.3%. Patients in State 3 tended to stabilize in State 2 (65.7%), whereas those in State 4 showed an increased probability of transitioning to State 5 (death) at 6.0%.

Table 5 demonstrates the mean sojourn times across the non-absorbing states. The estimated mean sojourn times illustrate that moderate symptoms persist longer, with patients remaining in the moderate state for around 11.45 days on average. Relative to this state, the mean sojourn time is shorter in State 4, estimated at 4.225 days, and in State 1, averaging 5.948 days.

**Table 5:** Estimated mean sojourn times of non-absorbing states

	Estimates	SE	L	U
State 1	5.948	0.240	5.495	6.438
State 2	11.449	0.271	10.931	11.992
State 3	4.738	0.186	4.388	5.116
State 4	4.225	0.832	2.873	6.214

Estimating transition probabilities and mean sojourn times reveals insights into the progression of COVID-19. After 1 week, patients with mild symptoms were more likely to progress to a moderate state, while those in moderate states tended to remain stable. A consolidation pattern emerged over 2 to 4 weeks, particularly among those initially presenting with mild or moderate symptoms, with a significant shift toward moderate conditions. However, for patients in severe or critical states, the probability of death increased notably over time, highlighting the urgent need for close monitoring and timely intervention in these high-risk groups.

### Effect of comorbidities on transitions

The results presented in Table 6 highlight the impact of comorbidities, specifically hypertension and diabetes, on the severity and progression of COVID-19. For individuals with hypertension, the hazard ratio for the transition from State 2 to State 3 was 1.676 (95% CI 1.445–1.944), indicating a 67.6% increased likelihood of aggravating from moderate to severe symptoms compared to those without hypertension. In the case of diabetes, the hazard ratio for transitioning from State 2 to State 3 was 1.799 (95% CI 1.518–2.131), indicating a 79.9% higher likelihood of developing severe symptoms. Furthermore, the hazard ratio for the transition from State 3 to State 4 is 1.899 (95% CI 1.023, 3.525), revealing that individuals with diabetes are 89.9% more likely to deteriorate to a critical state compared to those without diabetes. These findings underscore the influence of comorbidities such as hypertension and diabetes in exacerbating the clinical course of COVID-19, significantly increasing the risk of progression to more severe and critical states.

**Table 6:** Transition intensities with hazard ratios for covariates diabetes and hypertension

Transition	Baseline transition rate	Hypertension (HR)	Diabetes (HR)
State 1–State 1	− 0.166 (− 0.18, − 0.153)		
State 1–State 2	0.166 (0.153,0.18)	1.142 (0.961,1.357)	0.98 (0.794,1.21)
State 1–State 5	1.66e^−05^ (4.124 e−09,0.067)	0.664 (6.452e^−09^,6.832e^07^)	0.803 (1.036e^−10^,6.223e^09^)
State 2–State 1	0.046 (0.044,0.049)	1.229 (1.062,1.423)	1.162 (0.966,1.398)
State 2–State 2	− 0.086 (− 0.09, − 0.082)		
State 2–State 3	0.04 (0.037,0.042)	1.676 (1.445,1.944)	1.799 (1.518,2.131)
State 2–State 5	3.234e^−05^ (1.921e^−06^, 5.444e^−04^)	0.227 (4.941e^−05^,1.045e^+03^	0.4431 (1.427e^−05^,1.376e^+04^)
State 3–State 2	0.195 (0.18, 0.212)	1.038 (0.875,1.23)	0.9553 (0.783,1.165)
State 3–State 3	− 0.208 (− 0.225, − 0.192)		
State 3–State 4	0.001 (0.008, 0.017)	1.6 (0.881,2.909)	1.8989 (1.023,3.525)
State 3–State 5	7.829e^−05^ (4.422e^−07^, 0.013)	2.132 (0.222,2.049e^+01^)	84.999 (1.882e^−01^, 3.838e^+04^)
State 4–State 3	0.223 (0.143, 0.349)	0.722 (0.324,1.609)	1.717 (0.745,3.955)
State 4–State 4	− 0.227 (− 0.354, − 0.144)		
State 4–State 5	2.865e^−03^ (2.396e^−05^, 0.342)	10.4755 (1.224e^−02^,8.968e^+03^)	0.26 (8.642e^−05^, 7.839e^+02^)

## Discussion

This study utilized continuous-time Markov multistate models to investigate the progression dynamics of COVID-19 among hospitalized patients. The analysis focused on patient transitions within the first 2 weeks of hospitalization and revealed that most patients remained in their initial health state, indicating a general stabilization during the early stages of hospitalization. Notably, patients classified in State 2 exhibited the highest frequency of stabilization compared to all other severity states. Furthermore, patients in higher severity states showed a tendency to improve to lower severity states rather than deteriorate. Specifically, the likelihood of improving from State 3 to State 2 was found to be greater than the likelihood of worsening to State 4. These results indicate a trend toward recovery rather than symptom aggravation among the patients in this cohort. This trend can be attributed to the Korean government's effective and rapid response during the early phase of the pandemic, which prioritized widespread screening to detect the virus before it progressed to high-risk stages of severity. In the South Korean healthcare system, patients diagnosed with COVID-19 were classified based on the severity of their symptoms and treated accordingly [37]. Additionally, treatment strategies focused on identifying high-risk patients, particularly those over 60 years of age, individuals with diabetes, and those with weakened immune systems, prioritizing them for hospital care [38]. The timely implementation of vaccination in South Korea likely further contributed to the increased recovery rates observed among COVID-19 patients [39, 40]. These coordinated strategies were pivotal in achieving the high recovery rates observed in the Korean population, including the cohort analyzed in this study.

The incidence of hypertension and diabetes among infected patients was found to influence the aggravation of symptoms of COVID-19. Hypertension increases the likelihood of transitioning from moderate to severe disease states, while diabetes escalates the risk of aggravating from severe to critical conditions. Several mechanisms may contribute to the increased severity, including the upregulation of angiotensin-converting enzyme (ACE2), the receptor through which SARS-CoV-2 enters the host cell [41]. Both hypertension and diabetes, particularly when treated with ACE inhibitors or angiotensin II receptor blockers (ARBs), are associated with elevated ACE2 expression, potentially facilitating enhanced viral entry and replication. Additionally, diabetes has been found to impair immune responses and promote a pro-inflammatory state, further exacerbating the severity of COVID-19 [42]. These factors contribute to the heightened vulnerability of patients with diabetes and hypertension to rapid disease progression and severe outcomes, highlighting the critical need for tailored interventions and rigorous monitoring in these high-risk populations.

This study has several limitations. First, our dataset includes only clinical and demographic information, lacking important details such as patients’ vaccination status and the specific SARS-Cov-2 strains involved. Consequently, we were unable to assess the potential impact of vaccination or the variations in disease progression due to different viral strains. Second, this study utilized data exclusively collected during the hospitalization of COVID-19 patients. Due to the unavailability of pre-admission records, the dataset does not include information on patients' health conditions before hospitalization, including the period between the onset of symptoms and hospital admission. This limitation restricts our ability to evaluate the onset and progression of COVID-19 symptoms, which may influence the severity of the disease at the time of admission. Additionally, the study did not capture the time elapsed between infection and hospital admission. As patients were admitted at varying time points following infection, this introduces variability in disease progression that could not be fully accounted for in the analysis. Additionally, the data were collected from one single hospital in South Korea, which may limit the generalizability of our findings to a broader, global population. Future studies should aim to incorporate more diverse datasets from multiple regions and healthcare systems to enable a more comprehensive analysis that accounts for vaccination status and the evolving nature of the pandemic. Additionally, while this study employed continuous-time Markov multistate models, future research could also explore discrete-time Markov multistate models, which are particularly effective when data is collected at regular intervals, such as daily or weekly assessments. Discrete-time models facilitate the analysis of transition probabilities over fixed periods, offering valuable insights into the timing and patterns of disease progression. By integrating both continuous and discrete-time modeling approaches, future studies can provide a more nuanced understanding of COVID-19 progression and recovery dynamics, ultimately contributing to the refinement of global treatment and management strategies.

## Conclusion

This study employed continuous-time Markov multistate models to analyze the longitudinal progression and recovery dynamics of COVID-19 among hospitalized patients in a Korean cohort. The findings indicate that patients often stabilize in their initial health state during the early days of hospitalization, with a greater tendency for recovery than deterioration. Transition intensities show a high likelihood of mild symptoms worsening to moderate, yet recovery potential remains even in severe cases. The analysis highlights the importance of continuous monitoring and timely intervention, especially for patients in severe and critical states, while also demonstrating that comorbidities such as hypertension and diabetes significantly influence disease progression. Overall, this study underscores the need for proactive and individualized management strategies to effectively address the diverse progression patterns of COVID-19, particularly in high-risk patients, ultimately enhancing patient outcomes.

## Supplementary Information

Supplementary Table 1: Classification of COVID-19 Severity according to WHO.

## Data Availability

The dataset for this study is available on request to the corresponding author.

## References

[1] Wu F, et al. A new coronavirus associated with human respiratory disease in China. Nature. 2020;579(7798):265–9. 32015508 10.1038/s41586-020-2008-3 PMC7094943

[2] Gorbalenya A, et al. The species severe acute respiratory syndrome-related coronavirus: classifying 2019-nCoV and naming it SARS-CoV-2. Nat Microbiol. 2020;5(4):536–44. 32123347 10.1038/s41564-020-0695-z PMC7095448

[3] Lu R, et al. Genomic characterisation and epidemiology of 2019 novel coronavirus: implications for virus origins and receptor binding. The lancet. 2020;395(10224):565–74. 10.1016/S0140-6736(20)30251-8 PMC7159086 32007145

[4] Group, W.B. World Development Report 2022. Chapter 1. The economic impacts of the COVID-19 crisis [Website] 2022 [cited 2024 20 July 2024]; Available from: https://www.worldbank.org/en/publication/wdr2022/brief/chapter-1-introduction-the-economic-impacts-of-the-covid-19-crisis.

[5] Naseer S, et al. COVID-19 outbreak: impact on global economy. Front Public Health. 2023;10:1009393. 36793360 10.3389/fpubh.2022.1009393 PMC9923118

[6] Dubey S, et al. Psychosocial impact of COVID-19. Diabetes Metab Syndr. 2020;14(5):779–88. 32526627 10.1016/j.dsx.2020.05.035 PMC7255207

[7] Kupcova I, et al. Effects of the COVID-19 pandemic on mental health, anxiety, and depression. BMC psychology. 2023;11(1):108. 37041568 10.1186/s40359-023-01130-5 PMC10088605

[8] Leclerc QJ, et al. What settings have been linked to SARS-CoV-2 transmission clusters?. Wellcome Open Res. 2020;5. 10.12688/wellcomeopenres.15889.1 PMC7327724 32656368

[9] Harrison AG, Lin T, Wang P. Mechanisms of SARS-CoV-2 transmission and pathogenesis. Trends Immunol. 2020;41(12):1100–15. 33132005 10.1016/j.it.2020.10.004 PMC7556779

[10] Cevik M, et al. Virology, transmission, and pathogenesis of SARS-CoV-2. bmj. 2020;371. 10.1136/bmj.m3862 33097561

[11] Lau H, et al. The association between international and domestic air traffic and the coronavirus (COVID-19) outbreak. J Microbiol Immunol Infect. 2020;53(3):467–72. 32299783 10.1016/j.jmii.2020.03.026 PMC7271256

[12] Eurosurveillance Editorial Team. Note from the editors: World Health Organization declares novel coronavirus (2019-nCoV) sixth public health emergency of international concern. Eurosurveillance. 2020;25(5):200131e. 10.2807/1560-7917.ES.2020.25.5.200131e PMC7014669 32019636

[13] Organization, W.H. WHO Director-General's opening remarks at the media briefing on COVID-19 - 11 March 2020. 2020 11 March 2020 [cited 2024 25 July 2024]; Available from: https://www.who.int/director-general/speeches/detail/who-director-general-s-opening-remarks-at-the-media-briefing-on-covid-19---11-march-2020.

[14] Organization, W.H. WHO COVID-19 dashboard. 2024 August 1 [cited 2024 August 1]; Available from: https://data.who.int/dashboards/covid19/cases?n=c.

[15] Baj J, et al. COVID-19: specific and non-specific clinical manifestations and symptoms: the current state of knowledge. J Clin Med. 2020;9(6):1753. 10.3390/jcm9061753 PMC7356953 32516940

[16] World Health Organization. Clinical management of COVID-19: interim guidance, 27 May 2020. World Health Organization; 2020. https://iris.who.int/handle/10665/332196.

[17] Davies NG, et al. Age-dependent effects in the transmission and control of COVID-19 epidemics. Nat Med. 2020;26(8):1205–11. 32546824 10.1038/s41591-020-0962-9

[18] Guan W-J, et al. Clinical characteristics of coronavirus disease 2019 in China. N Engl J Med. 2020;382(18):1708–20. 32109013 10.1056/NEJMoa2002032 PMC7092819

[19] Wu, Z. and J.M. McGoogan, Characteristics of and important lessons from the coronavirus disease 2019 (COVID-19) outbreak in China: summary of a report of 72 314 cases from the Chinese Center for Disease Control and Prevention. jama. 2020;323(13):1239–1242. 10.1001/jama.2020.2648 32091533

[20] Das AK, Mishra S, Gopalan SS. Predicting CoVID-19 community mortality risk using machine learning and development of an online prognostic tool. PeerJ. 2020;8:e10083. 33062451 10.7717/peerj.10083 PMC7528809

[21] Saadatmand S, et al. Using machine learning in prediction of ICU admission, mortality, and length of stay in the early stage of admission of COVID-19 patients. Ann Oper Res. 2023;328(1):1043–71. 10.1007/s10479-022-04984-x PMC9521862 36196268

[22] Wu Y, et al. A deep learning method for predicting the COVID-19 ICU patient outcome fusing X-rays, respiratory sounds, and ICU parameters. Expert Syst Appl. 2024;235:121089.

[23] Singh RK, Pandey R, Babu RN. COVIDScreen: explainable deep learning framework for differential diagnosis of COVID-19 using chest X-rays. Neural Comput Appl. 2021;33(14):8871–92. 33437132 10.1007/s00521-020-05636-6 PMC7791540

[24] Singh T, et al. COVID-19 severity detection using chest X-ray segmentation and deep learning. Sci Rep. 2024;14(1):19846. 39191941 10.1038/s41598-024-70801-z PMC11349901

[25] Ge E, et al. Association of pre-existing comorbidities with mortality and disease severity among 167,500 individuals with COVID-19 in Canada: A population-based cohort study. PLoS ONE. 2021;16(10):e0258154. 34610047 10.1371/journal.pone.0258154 PMC8491945

[26] Singh P, et al. Impact of comorbidity on patients with COVID-19 in India: a nationwide analysis. Front Public Health. 2023;10:1027312. 36777781 10.3389/fpubh.2022.1027312 PMC9911546

[27] Noh E, et al. Inference and forecasting phase shift regime of COVID-19 sub-lineages with a Markov-switching model. Microbiology Spectrum. 2023;11(6):e01669-e1723. 37811981 10.1128/spectrum.01669-23 PMC10714866

[28] Wang C, Mustafa S. A data-driven Markov process for infectious disease transmission. PLoS ONE. 2023;18(8):e0289897. 37561743 10.1371/journal.pone.0289897 PMC10414655

[29] Hazard D, et al. Joint analysis of duration of ventilation, length of intensive care, and mortality of COVID-19 patients: a multistate approach. BMC Med Res Methodol. 2020;20(1):206. 32781984 10.1186/s12874-020-01082-z PMC7507941

[30] Al-Zoughool M, et al. Using a stochastic continuous-time Markov chain model to examine alternative timing and duration of the COVID-19 lockdown in Kuwait: what can be done now? Archives of Public Health. 2022;80(1):22. 34998438 10.1186/s13690-021-00778-y PMC8742165

[31] Mude W, et al. Racial disparities in COVID-19 pandemic cases, hospitalisations, and deaths: A systematic review and meta-analysis. J Glob Health. 2021;11. 10.7189/jogh.11.05015 PMC8248751 34221360

[32] Dergiades T, et al. Effectiveness of government policies in response to the first COVID-19 outbreak. PLOS Global Public Health. 2022;2(4):e0000242. 36962226 10.1371/journal.pgph.0000242 PMC10021334

[33] Escobedo-de la Peña, J., et al., Hypertension, diabetes and obesity, major risk factors for death in patients with COVID-19 in Mexico. Archives of medical research. 2021; 52(4):443–449. 10.1016/j.arcmed.2020.12.002 PMC7832055 33380361

[34] Albitar O, et al. Risk factors for mortality among COVID-19 patients. Diabetes Res Clin Pract. 2020;166:108293. 32623035 10.1016/j.diabres.2020.108293 PMC7332436

[35] Du P, et al. A systematic review and meta-analysis of risk factors associated with severity and death in COVID-19 patients. Canadian Journal of Infectious Diseases and Medical Microbiology. 2021;2021(1):6660930. 10.1155/2021/6660930 PMC8040926 33936349

[36] Jackson C. msm: Multi-state Markov and hidden Markov models in continuous time. R package version 0.8 1. 2008.

[37] You J. Lessons from South Korea’s Covid-19 policy response. The American Review of Public Administration. 2020;50(6–7):801–8.

[38] Oh J, et al. National response to COVID-19 in the Republic of Korea and lessons learned for other countries. Health Systems & Reform. 2020;6(1):e1753464. 32347772 10.1080/23288604.2020.1753464

[39] Yi S, et al. Impact of national Covid-19 vaccination campaign. South Korea Vaccine. 2022;40(26):3670–5. 35570077 10.1016/j.vaccine.2022.05.002 PMC9080122

[40] Kwon SL, Oh J. COVID-19 vaccination program in South Korea: a long journey toward a new normal. Health policy and technology. 2022;11(2):100601. 35127400 10.1016/j.hlpt.2022.100601 PMC8801590

[41] Fang L, Karakiulakis G, Roth M. Are patients with hypertension and diabetes mellitus at increased risk for COVID-19 infection? Lancet Respir Med. 2020;8(4):e21. 32171062 10.1016/S2213-2600(20)30116-8 PMC7118626

[42] Bornstein SR, et al. Practical recommendations for the management of diabetes in patients with COVID-19. Lancet Diabetses Endocrinol. 2020;8(6):546–50. 10.1016/S2213-8587(20)30152-2 PMC7180013 32334646

