# Supplementary material for: Analyzing COVID-19 progression with Markov multistage models: insights from a Korean cohort
Source: Genomics Inform. 2025 Jan 27;23:2. doi: 10.1186/s44342-024-00035-y (PMC11786383; doi:10.1186/s44342-024-00035-y)
Supplement: Supplementary file 1 — Supplementary Table 1: Classification of COVID-19 Severity according to WHO. [file 44342_2024_35_MOESM1_ESM.docx]

**Supplementary Table 1: Classification of COVID-19 Severity according to WHO**

| **Severity state** | **Description** | **Characteristics** |
| --- | --- | --- |
| State 1 | Mild illness | Individuals with fever, cough malaise, headache, muscle pain, nausea, vomiting, diarrhea, loss of taste and smell  However, the patient does not have shortness of breath, dyspnea, or abnormal chest imaging |
| State 2 | Moderate illness | Individuals who show evidence of lower respiratory disease during clinical assessment or imaging and who have an oxygen saturation measured by pulse oximetry (SpO_2_) ≥ 94% on room air at sea level. |
| State 3 | Severe illness | Individuals who have an SpO2 <94% on room air at sea level, a ratio of arterial partial pressure of oxygen to fraction of inspired oxygen (PaO2/FiO2) <300 mm Hg, a respiratory rate >30 breaths/min, or lung infiltrates >50%. |
| State 4 | Critical illness | Individuals who have respiratory failure, septic shock, or multiple organ dysfunction. |
| State 5 | Death | |
